# Taxonomic Analysis of Some Edible Insects From the State of Michoacán, Mexico

**DOI:** 10.3389/fvets.2021.629194

**Published:** 2021-03-04

**Authors:** José Manuel Pino Moreno, Julieta Ramos-Elorduy Blasquez

**Affiliations:** Entomology Laboratory, Department of Zoology, Biology Institute, National Autonomous University of Mexico, Mexico City, Mexico

**Keywords:** entomophagy, Mexico, Michoacán, taxonomy, gastronomy

## Abstract

In the state of Michoacán, Mexico, 49 genera and 69 species of edible insects were registered, and they belong to the following orders: Orthoptera: (families) Pyrgomorphidae (2 species) and Acrididae (5); Hemiptera-Heteroptera: Coreidae (1), Corixidae (2), Pentatomidae (2), Membracidae (2), and Aethalionidae (3); Coleoptera: Cerambycidae (1), Cicendelidae (2), Curculionidae (2), Dytiscidae (1), Noteridae (1), Gyrinidae (1), Passalidae (1), Scarabaeidae (1), Tenebrionidae (2), Bostrichidae (1), Buprestidae (1), and Melolonthidae (1); Lepidoptera: Cossidae (1), Danaidae (1), Megathymidae (1), Pieridae (1), Bombycidae (1), Sessidae (1), Noctuidae (1), and Nymphalidae (1); Diptera: Stratiomydae (2); and Hymenoptera: Diprionidae (2), Apidae (10), Formicidae (4), and Vespidae (12). The order Coleoptera presents the highest number of families (12), but the order Hymenoptera has the highest number of genera (18) and species (28), 12 of which belong to the family Vespidae. Among the genera, 75.52% are monospecific, 14.28% are bispecific, 4.08% are trispecific, 4.08% are tetraspecific, and 2.04% are pentaspecific. Their distribution by locality is indicated, and these localities correspond to the municipalities of the state sampled so far; likewise, we report various forms in which they are prepared and the economic importance of, for example, the grasshoppers of the genus *Sphenarium* that are widely looked for, collected, accepted as part of the diet, consumed, and marketed.

## Introduction

Society currently faces a variety of significant crises. Preexisting quotidian crises (such as currency devaluation, rising product prices, overproduction of processed foodstuffs, drought, floods, etc.) are now accompanied by drastic climate fluctuations caused by global warming, including issues such as rising pollution, unchecked demographic growth, and low crop yield. As a result, to feed an ever-growing world population, insects are an important alimentary resource. Not only do many animals feed on them, but they also have a long culinary history providing food for people in different parts of the world: these animals have been valued by many cultures and for some are the sole protein source available (1). Being a regular component of the diet of many countries, almost 1,900 species are consumed by 3,000 cultural groups in 13 cities; in the order of importance due to the number of species that are widely consumed, lepidopters, hymenopterans, orthopters, bugs, cockroaches, dragonflies, and flies are consumed in the developmental stages known as eggs, larvae, pupae, and adults (2). As our attention mostly focuses on increasing the production of foodstuffs that must be abundant, innocuous, and nutritious, with a good rate of energy turnover, diverse international agencies such as the FAO (2) have proposed that insects may solve this problem, and there is evidence that people of different social backgrounds look for and consume insects in countries like Argentina, Australia, Brazil, Colombia, Costa Rica, China, Ecuador, Spain, United States, France, India, Kenya, Japan, Mexico, Thailand, and Venezuela (2–7). Insects are the primary alimentary source of all terrestrial and freshwater ecosystems; for example, spiders, scorpions, fish, amphibia, reptiles, birds, and mammals survive in the trophic chains, thanks to insect consumption, and insects are also bred as feed for tarantulae, fish, reptiles, poultry, and diverse types of pets to which these arthropods provide balanced quantities of nitrogen, phosphorus, potassium, calcium, and also calories (1, 8). Insects constitute the largest biomass on the planet, and societies have used them to obtain stains, foodstuffs, medicines, honey, silk, lacquer, fertilizers, etc. throughout history (9–12). At present, it is widely known that insects harbor a high mineral nutritious value. They are relatively high in fat and rich in protein, B group vitamins, and energy (13–17); they also contain biodynamic compounds important for human health (18) and have a caloric yield rate, among other nutritional properties, much higher than that of vertebrates (19).

The state of Michoacán is situated on the volcanic axis in part of the Balsas and the Sierra Madre del Sur basin. Its limits extended to Jalisco and Guanajuato in the north, Querétaro in the northwest, Estado de México in the east, Guerrero in the south and southeast, the Pacific Ocean in the south and southwest, and Colima and Jalisco in the west; it covers an area of 59,928 km^2^. The topography of the state include Sierra Tarasca in the north pertaining to the Eje Volcánico along with numerous volcanoes, such as Zacapú; the main mountain ranges such as the ones of Angangueo in the limits of the Estado de México; Ucareo, Mil Cumbres, and Otzumatlán; the Pico de Orizaba; mount Zirate; the Sierra de Patambán; mount Tancítaro; Sierra de Inguarán; the Paricutín and Jorullo volcanoes; and the northern part of the Río Tepalcatepec basin contained the flatlands of Antunez, Lombardia, and Nueva Italia. Sierras Tarasca and Coalcamán have a subhumid temperate climate, and the mountainsides toward the basins of Tepalcatepec and Balsas and toward the Pacífic are hot humid and the lower part of that basin is semi-dry with a summer rainfall regime. The following rivers are present in the state: Coahuayana, Coire, Coalcamán, Nexpa, Carrizal, and Zacatula; the last one has numerous tributaries in the state, such as the Grande, which also receives water from the Cupatitzio or del Marqués and forms the waterfall known as the Tzaráracua at the south of the city of Uruapán, the Tacámbaro, the Carácuaro, the Cutzamala, the Tuzantla, the Tuxpan, the Zitácuaro, and the Temascaltepec. The river Lerma marks boundaries between Michoacán and the states of Querétaro, Guanajuato, and Jalisco; the closed basins include lakes Cuitzeo, Pátzcuaro, and Zirahuén. The state also has numerous springs of thermal and mineral-medicinal waters. In the Sierra Madre del Sur, the soils are poor and undeveloped, such as the regosols and cambisols; in the Sierra Tarasca, the soils are fertile and derive from volcanic ash (andosols); and in the Balsas basin, the soils are clayey lateritic (acrisol and luvisol) alternating with cambisol. The state is divided into 113 municipalities and 7,716 localities. Of the total area of the state, 33.3% is farmland, 30.4% is rainfed, and 4.9% is irrigated, that is, Michoacán is an agricultural state that produces corn, sorghum, rice, beans, wheat, barley, safflower, sesame, sugar cane, cotton, alfalfa, potatoes, and tomatoes and fruits such as strawberries, melons, avocados, lemons, mangos, apples, watermelons, and bananas. Grasslands compromise 6.7% of the total state area. Bovine cattle are the most important type of livestock, significant of which were the porcine, along with goats, sheep, horses, mules, and donkeys being bred. The total forest area is of 4,320,800; 2,052,800 ha are covered in trees, and of these, 1,733,200 ha has temperate and semicold climate forests and 319,000 medium forests, low forests, and mezquital correspond to 867,600 ha, shrubland to 259,200 ha and the areas that have been cleared so as to be used otherwise represent 1,141,200 ha. Pines, oaks, and firs are exploited for their wood; resin is obtained from pines. Fishing activities in its lakes are also important for its economy; the main species are charal, carp, catfish, frog, white fish, huachinango, tilapia, and sardine (20).

Currently, many countries are seeking sustainable ways to use and preserve landscapes. It is very important to make an inventory of edible insects, whilst also respecting conservation and finding ways to ensure the rational management of biodiversity, which is critical for human survival. This is why, in this case, the objective of this study was to carry out exploratory research so as to know, collect, and identify the edible insect diversity in the state of Michoacán, Mexico, as well as discuss the economic and gastronomic importance of some of the species registered.

## Materials and Methods

### Field Work

During 2017 and 2019, diverse visits to 48 localities were carried out so as to survey and collect the edible insects. With this in mind, we developed a questionnaire exclusively aimed at the inhabitants of rural areas in which we asked about the insects included in their diet, their common names and hosts, and their gastronomic importance. Edible insects were collected using tweezers and entomological nets (aerial, aquatic, and sweep nets); they were then placed in plastic jars filled with 70% ethanol for their preservation. In all the samples collected, we included the following data: locality name, collection date, collector's name, common name, and type of host (21). Several insects were bought in some *tianguis* (small markets, mainly the ones that are installed periodically on the street) and/or municipal markets.

### Laboratory activities

After their collection, insects were brought to the Entomology Laboratory that is part of the Department of Zoology of the Biology Institute, where they were mounted, labeled (21), and identified by means of the taxonomical keys available for the different orders, for example, Orthoptera: (22–24); Hemiptera-Heteroptera: (25–34); Coleoptera: (35–37); Lepidoptera: (38, 39); Diptera: (40) and Hymenoptera: (41–44). Afterwards, they were ratified by comparing them with the insect collection and the aid of different experts from the Biology Institute of the National Autonomous University of Mexico so as to be finally cataloged and introduced in the edible insect collection that is part of the National Insect Collection of the same institute. We also report edible insects recorded in the literature of other authors such as Argueta and Castilleja (45), Lagunas (46), Reyes et al. (47), and Reyes et al. (48).

In alphabetical order, the localities sampled were: Ahuiran, Alto Balsas, Angangueo, Aquila, Aranza, Ario de Rosales, Capacuaro, Angahuan, Chauzingo, Charapan, Cherán, Cocucho, Copándaro de Galeana, Cotija, Eronganicuaro, Jerecuaro, Jiquilpan, Juchitán, Jungapeo, La Piedad, Lago de Cuitzeo, Lago de Pátzcuaro, Las Cocinas, Mazamitla, Meseta Tarasca, Morelia, Nahuatzen, Neocupétaro, Pátzcuaro, Pomocuaran, Quinceo, San Francisco Corupo, San Lorenzo, San Pedro Tarimbaro, Sevina, Tacámbaro, Tecomán, Tingambato, Tlalpujahua, Tumbizca, Tupataro, Tuxpan, Uruapan, Zacán, Zamora, Zirahuen, Zitácuaro, and Ziracuaretiro. The collection sites include pine-oak forests, ravines, shrubland, hills, and springs.

## Results and Discussion

### Taxonomic Analysis

The edible insects reported by the persons interviewed are presented in **Table 2**.

In this study, we report 6 orders, 31 families, 49 genera, and 69 species.

**Graph 1:**
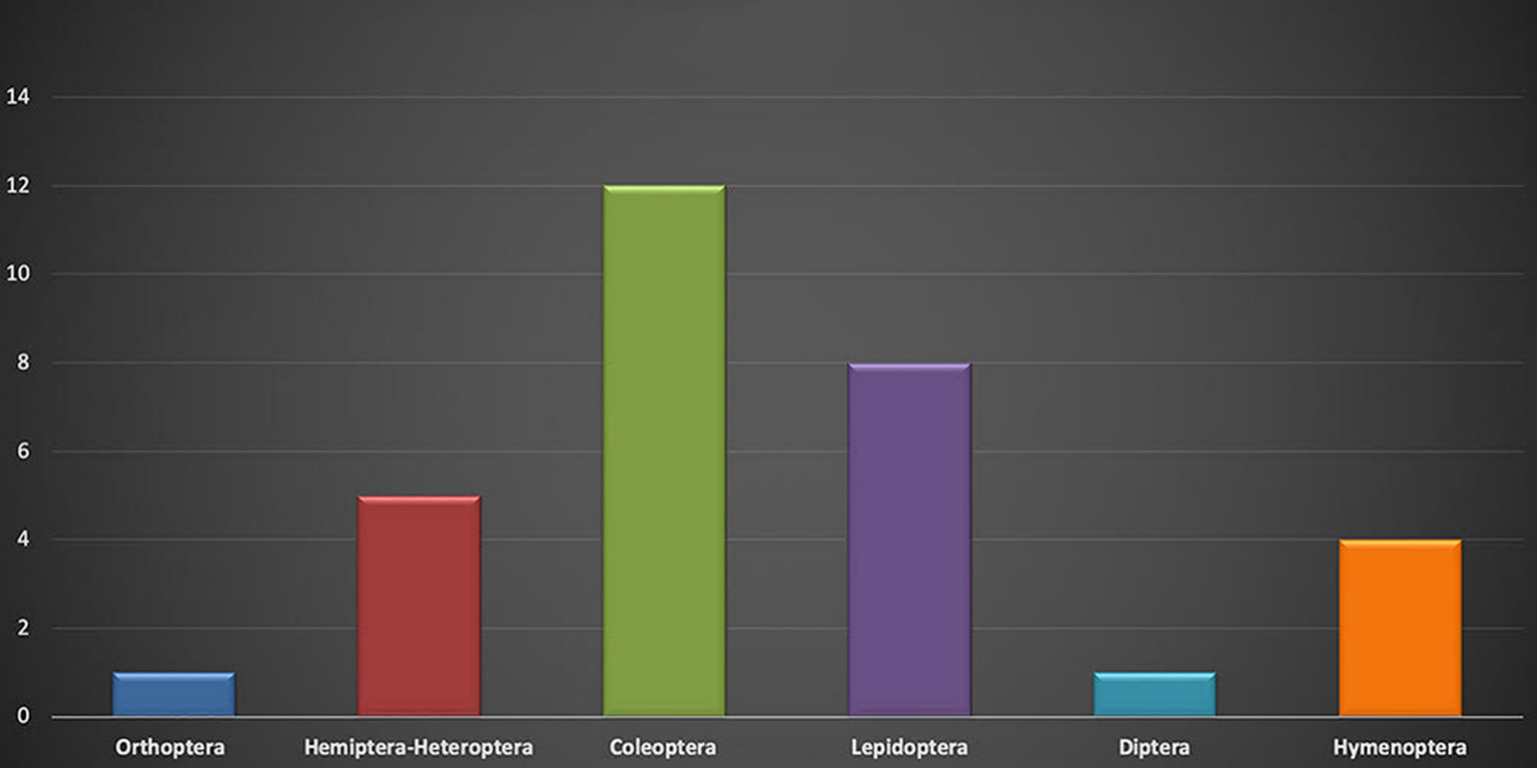
Number of families per order.

The order Coleoptera has the highest number of families (13) (Cerambycidae, Cicindelidae, Curculionidae, Dytiscidae, Noteridae, Gyrinidae, Passalidae, Scarabaeidae, Tenebrionidae, Bostrichidae, Buprestidae, and Melolonthidae) (Graph 1). In this order, it is mainly the larvae that are consumed in the developmental stage; only in the case of *Trichoderes pini* are both larvae and pupae are eaten, and, in the genus *Cysbister*, the larvae and adults are eaten. It is convenient to point out that the larvae are the most digestible in the developmental stage as they possess the smallest quantity of “raw fiber” and that the adults are consumed in very few cases.

**Graph 2:**
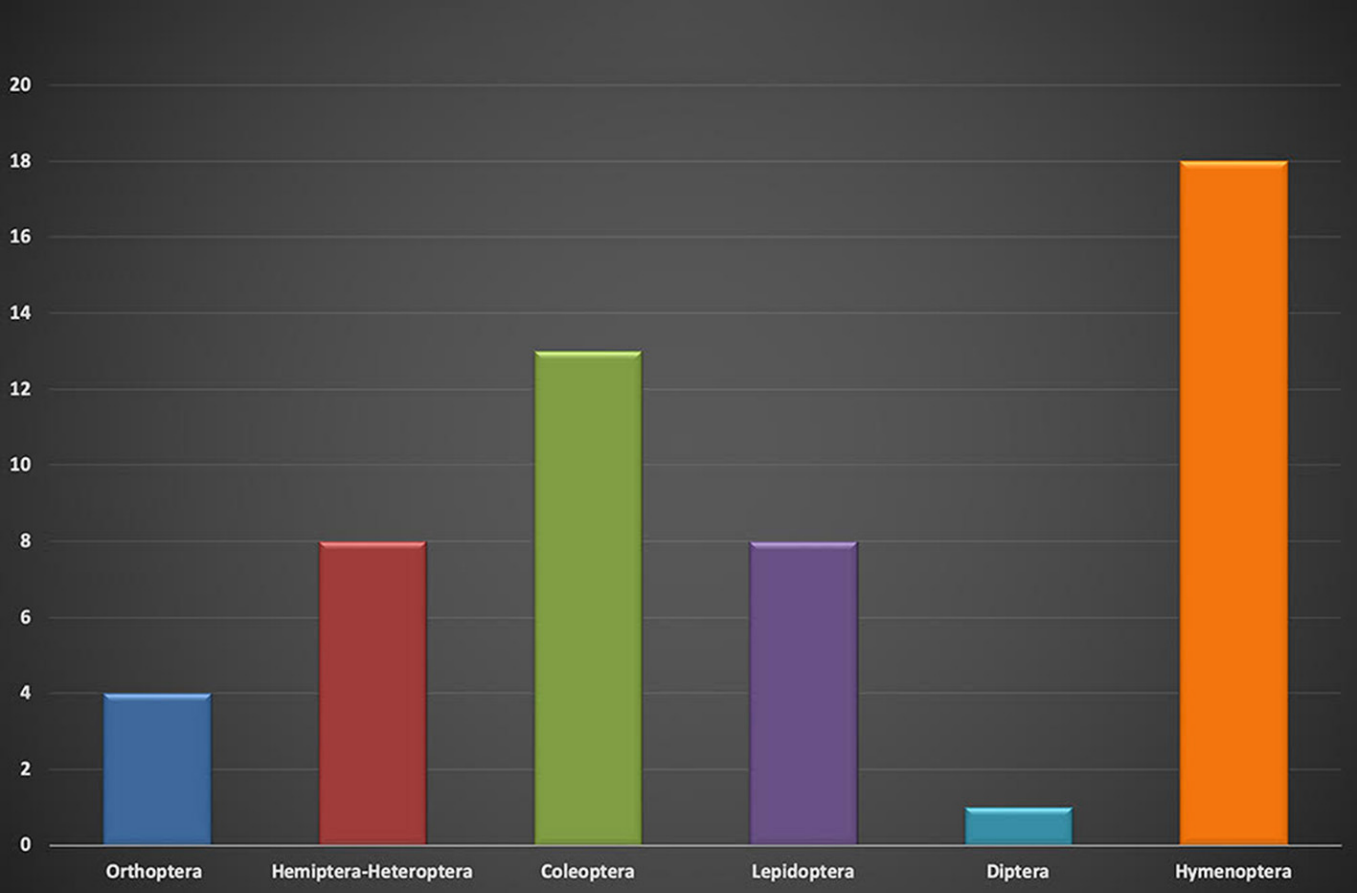
Number of genera per order.

**Graph 3:**
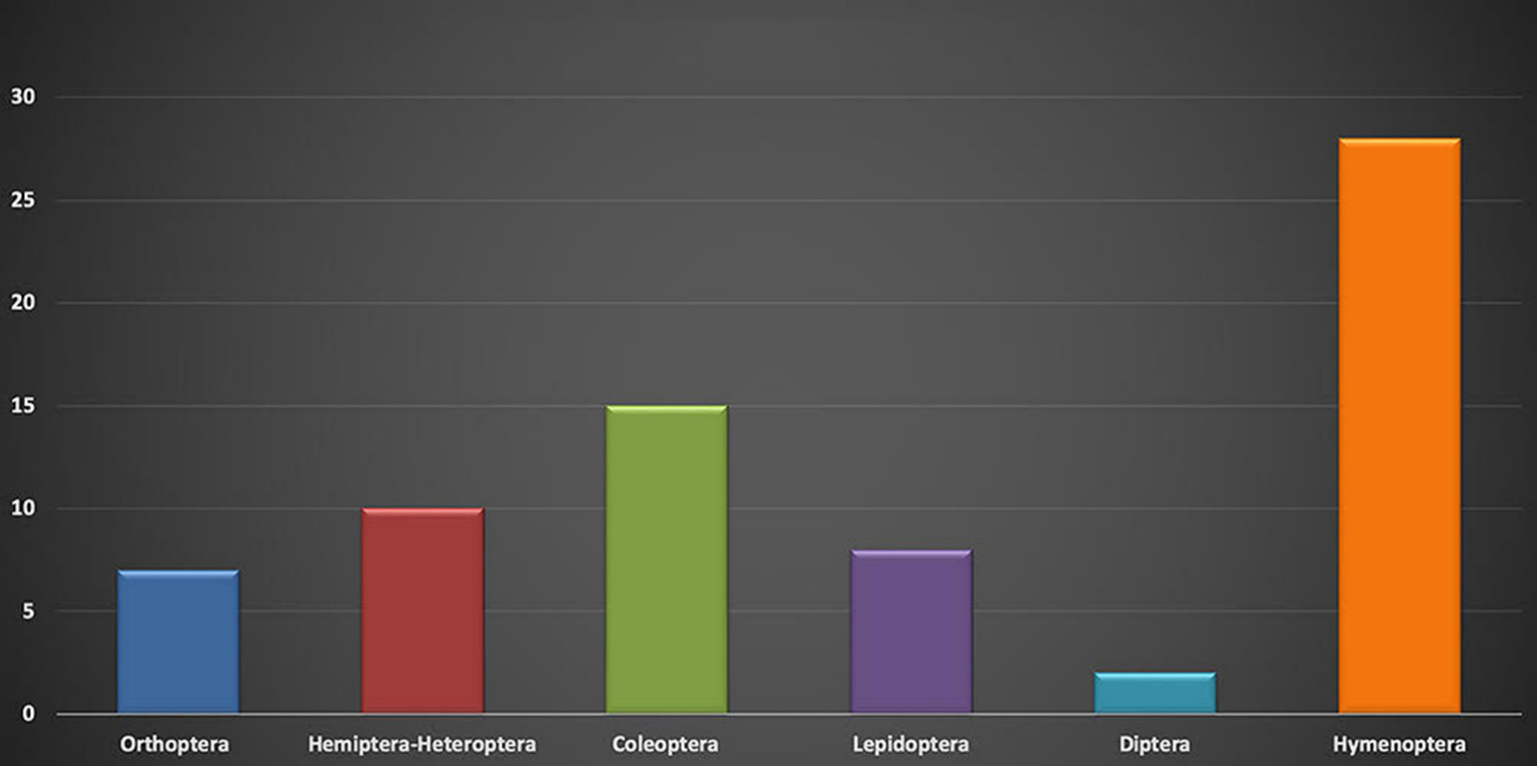
Number of species per order.

The order Hymenoptera has the highest number of genera (19) and species (28) (Graphs 2,3), and the family Vespidae holds the largest number of species (13). In hymenopterans, all in the immature developmental stages (eggs, larvae, and pupae) are generally consumed; in bees, both stingless bees and the genus *Polybia*, the consumption of honey is well-known and reproducing adults are consumed in the case of the ants of the genus *Atta*. A taxonomic synthesis is presented in Table 1.

**Table 1:** Taxonomic synthesis of the number of families, genera, and species in order.

	**Families**	**Genera**	**Species**
Orthoptera	1	4	7
Hemiptera-Heteroptera	5	8	10
Coleoptera	12	13	15
Lepidoptera	8	8	8
Diptera	1	1	2
Hymenoptera	4	18	28

We also record common or linguistic names, where the edible stage of development and the localities in which the consumption was recorded (Table 2).

**Table 2:** Taxonomic list of the edible insects of the state of Michoacán.

**Order–family**	**Genus**	**Species**	**Common or linguistic name**	**Edible stage**	**Place of consumption**
**Orthoptera:** Acrididae	*Melanoplus*	*differentialis* (Thomas, 1865)	Grasshopper	N, A	Jungapeo
	*Spharagemon*	*equale* (Say, 1825)	Grasshopper	N, A	Jungapeo
	*Orphulella*	*orizabae* (Mcneill, 1897)	Grasshopper	N, A	Jungapeo
	*Orphulella*	*tolteca* (Saussure, 1861)	Grasshopper	N, A	Jungapeo
	*Orphulella*	*quiroga* (Otte, 1979)	Grasshopper	N, A	Uruapan
	*Sphenarium*	sp.	Grasshopper	N, A	Las Cocinas, Charapan, Nahuatzen
	*Sphenarium*	*purpurascens* (Charpentier, 1842)	Grasshopper	N, A	Charapan
**Hemiptera-Heteroptera:** Coreidae	*Thasus*	*gigas* (Klug, 1835)	“Xamues,” “Cocopaches”	N, A	Copándaro de Galeana
Corixidae	*Corisella*	*mercenaria* (Say, 1832)	“Mosco,” “Axayacatl,” “Ahuahutle”	E, N, A	Lago de Cuitzeo
	*Krizousacorixa*	*femorata* (Guerin-Meneville, 1844)	“Mosco,” “Axayacatl,” “Ahuahutle”	E, N, A	Lago de Cuitzeo
Pentatomidae	*Brochymena (Arcana)*	*tenebrosa* (Walker, 1867)	“Jumil”	N, A	Cotija
	*Chlorocoris*	sp.	“Jumil”	N, A	Copándaro de Galeana
Membracidae	*Hoplophorion (Metcalfiella)*	*monograma* (Germar, 1835)	“Periquito del aguacate”	N, A	Juchitán, Uruapan, Jiquilpán
	*Stictocephala*	*bisonia* (Koop Yonke, 1977^1^)	–	N, A	–
Aethalionidae	*Aethalion*	*quadripunctatus*	–	N, A	Uruapan
	*Aethalion*	*nervosum punctatum* (Signoret, 1851)	–	N, A	Jiquilpán
	*Aethalion*	*quadratum* (Fowler, 1897)	Avocado greenfly	N, A	Tingambato
**Coleoptera:** Cerambycidae	*Arhopalus*	sp.	Pine worm	L, P	Charapan, San Francisco Corupo, Pomocuaran, Zacán, Aranza
Cicindelidae	*Cicindela (grupo dorsalis)*	*curvata*	–	L	Zitácuaro
	*Cicendela (grupo rufiventris)*	*roseiventris* (Chevrolat, 1834)	–	L	Tacambaro
Curculionidae	*Rhynchophorus*	*palmarum* (Linnaeus, 1758)	Coconut palm weevil	L	Tecoman
	*Sitophilus*	sp.	Corn weevil	L	Zacán, Ahuiran
Dytiscidae	*Cybister*	sp.	–	L, A	Pátzcuaro
Noteridae	*Suphisellus*	sp.	–	L, A	Zamora
Gyrinidae	*Gyrinus*	*parcus* (Say, 1834)	Whirlwind beetle	L	Tupataro
Passalidae	*Passalus (Passalus)*	*af. punctiger*	Rotten log worm	L	Mazamitla
Scarabaeidae	*Phyllophaga*	sp.	“Gallina ciega”	L	Quinceo, Pomocuaran Charapan, Tumbizca
Tenebrionidae	*Tenebrio*	sp.	Meal worm	L	Tuxpan
	*Tenebrio*	*molitor* (Linnaeus, 1758)	Yellow flour worm	L	Tuxpan
Bostrichidae	*Prostephanus*	*truncatus* (Horn, 1878)	Larger grain borer	L	Zacán, Ahuiran
Buprestidae	*Chalcophora*	sp.	Pine log worm	L	Charapan, Capacuaro, San Felipe de los Herreros
Melolonthidae	*Dynastes*	*hyllus* (Chervrolat, 1843)	Avocado trunk worms	L	Cotija, Uruapan
**Lepidoptera:** Cossidae	*Comadia*	*redtenbacheri* (Hammerschmidt, 1848)	Red maguey worm	L	San Pedro Tarimbaro
Danaidae	*Danaus*	*plexippus* (Linnaeus, 1758)	Monarch butterfly	L	Angangueo
Megathymidae	*Aegiale*	*hesperiaris* (Walker, 1856)	White maguey worm	L	Tlalpujahua, San Pedro Tarimbaro, Capacuaro, San Francisco Corupo, Ario de Rosales
Pieridae	*Eucheira*	*socialis* (Westwood, 1834)	Arbutus tree worm “cupiche”	L	San Pedro Tarimbaro, Aranza, Aquila, Charapan, Pomocuaran, Pátzcuaro, Zitácuaro
Bombycidae	*Bombyx*	*mori* (Linnaeus, 1758)	Silkworm	L	Ziracuaretiro
Sessidae	*Synanthedon*	*cardinalis* (Damf, 1930)	Resin moth	L	Charapan
Noctuidae	*Helicoverpa*	*zea* (Boddie, 1850)	Corn worm	L	Anganhuan, Zacán
Nymphalidae	*Charaxes*	*jasius* (Linnaeus, 1767)	“Cupiches,” “Huenches,” “Conduchas,” or “Chamas”	P	Zitacuaro, Patzcuaro
**Diptera:** Stratiomydae	*Hermetia*	*aurata* (Bellardi, 1859)	Soldier fly	L	Morelia
	*Hermetia*	*ceria* (Williston, 1900)	Soldier fly	L	Morelia
**Hymenoptera:** Diprionidae	*Neodiprion*	*guilletei*	Saw fly	E, L, P	Charapan, Meseta Tarasca
	*Zadiprion*	*falsus* (Smith, 1988) = *vallicola*	Saw fly	E, L, P	Charapan, Meseta Tarasca Cocinas, Angahuan, San Lorenzo
Apidae	*Apis*	*mellifera adansonii* (Latreille, 1804)	Honey bee	E, L, P, H	La Piedad
	*Lestrimelitta*	*chamelensis* (Ayala, 1999^2^)	Stingless bee	L, P, A. H^5^	Neocupétaro
	*Melipona*	*fasciata* (Latreille, 1811^2^)	Stingless bee	E, L, P H^4^	Neocupétaro
	*Scaptotrigona*	*hellwegeri* (Friese, 1900^2^)	Vermilion bee	L, P (H^4^) W	Neocupétaro
	*Trigona*	*nigra* (Cresson, 1878)	Stingless bee	E, L, P	Zacapu
	*Geotrigone*	*acapulconis* (Strand, 1919)	Ground hive	E, L, P H^5^	Neocupétaro
	*Plebeia*	*fulvopilosa* (Ayala, 1999^1, 2^)	“Abeja sapita”	NP	Alto Balsas
	*Trigonisca*	sp.^2^	“Abeja cepimilla”	H^1, 5^	Neocupétaro
	*Nannotrigona*	*perilampoides* (Cressson, 1878^2^)	“Abeja trompetera”	H^4^	Uruapan
	*Frisiomelitta*	*nigra* (Cresson, 1870^2^)	“Abeja zopilota”	H^4^	Neocupétaro
Formicidae	*Liometopum*	*occidentale var. luctuosum* (Emery, 1895)	“Escamol”	E, L, P	Tlalpujahua, San Pedro Tarímbaro
	*Liometopum*	*apiculatum* (Mayr, 1870)	“Escamol”	E, L, P	Tlapujahua, San Pedro Tarímbaro
	*Atta*	*mexicana* (Smith, 1858)	“Chicatanas,” “Nucú”	RA	Eronganicuaro, Tingambato
	*Atta*	*cephalotes* (Linnaeus, 1758)	“Chicatanas,” “Zampopo”	RA	Eronganicuaro, Tingambato
Vespidae	*Brachygastra*	*lecheguana* (Latreille, 1824)	–	E, L, P	Cheran
	*Polistes*	*instabilis* (Saussure, 1853)	“Avispa zapatona”	E, L, P	Jungapeo
	*Polistes*	*major* (Palisot de Beauvois, 1818)	–	E, L, P	Jungapeo
	*Polistes (Polisotius)*	*major major* (Palisot de Beauvois, 1818)	–	E, L, P	Zamora
	*Polistes*	sp.		E, L, P	Cherán
	*Polybia*	*occidentalis nigratella* (Buysson, 1915)	Juniper wasp	E, L, P	Morelia, Jerecuaro, Jungapeo, Lago de Pátzcuaro, Cheran
	*Polybia*	*occidentalis bohemani* (Holmgren, 1868)	“Avispa rayada”	E, L, P	Chauzingo, Morelia, Jerécuaro, Charapan, Meseta Tarasca, Sevina, Cheran, Aranza. Cocucho
	*Polybia*	*parvulina* (Richards, 1970)	Black wasp	E, L, P	Lago de Pátzcuaro
	*Polybia*	sp. (a)	–	E, L, P	San Pedro Tarimbaro, Cherán, Ahuiran, Nahuatzen
	*Polybia*	sp. (b)^3^	“Uauapu,” “moxquito pequeño,” “abejitas negras y pequeñas”	L, C, H, Ho	Sierra Tarasca
	*Vespula*	*pensylvanica* (Saussure, 1857)	–	E, L, P	Lago de Pátcuaro
	*Vespula*	*squamosa* (Drury, 1773)	Honeycomb ground	E, L, P	Aranza, Zirahuen, Cheran, Zacan Pomocuarán

1 *Reyes et al. (48)*,

2 *Reyes et al. (47)*,

3 *Villamar and Castilleja (45)*.

4 Alimentary and

5 *Medicinal. E, Eggs; L, Larvae; P, Pupae; A, Adults; RA, Reproductive adults; Ho, Honeycomb; H, Honey; W, Wax; NP, Nest products. Finally (a) and (b) are two species of the genus Polybia that are in the process of identification*.

Five species have an aquatic habit, that is, 7.24% (*Corisella mercenaria, Krizousacorixa femorata, Cybister* sp., *Suphisellus* sp., and *Gyrinus parcus*), and 92.76% are terrestrial.

As compared with other states in relation to the number of species recorded, Hidalgo has 99, Oaxaca 79, Guerrero 50, Estado de México 105, Chiapas 178, and Morelos 61 (49, 50). This means that the number of species is higher in Michoacán than in Guerrero and Morelos but lower than in Hidalgo, Estado de México, and Chiapas. This is most probably due to the diverse socio-economic activities carried out in the state, and among them are agriculture, animal husbandry, forestry, and fishing. Agriculture, for example, is the main activity, and it yields 84.9% of the Mexican production of avocado; in this case, this aspect can be seen as a means of diversifying foodstuffs. Insect collection and recording depends on factors such as abundance throughout the year, reproductive capacity, number of generations per year, and ecological traits of the sampled zones. The number of species so far recorded is significant and provides an aid for developing and rescuing and information that will help promote and preserve knowledge about this alimentary habit. The advantages of insect consumption by humans and/or animals are widely known, among them, for example, they form part of traditional habits; they are innocuous; they are rich in proteins, essential and non-essential amino acids, saturated and unsaturated fatty acids, minerals such as calcium, iron, and zinc, and vitamins A, D, and C (14, 51–54); they possess a satisfactory efficiency of food conversion (55) which is being able to feed on organic detritus (11); and they discharge a smaller quantity of greenhouse gases as compared to, for example, poultry production, and other animal husbandry.

Their culture requires a smaller area and a minimum proportion of feed, their collection and culture require a minimum of infrastructure and investment, and they offer employment opportunities for people in both rural and urban areas (2). Even today they are asked for, looked for, collected, produced in the sustainable alimentary systems, commercialized, and prepared by many chefs in diverse countries worldwide as snacks, appetizers, desserts, and in gourmet dishes, all of which is evidence of the acceptance they have and their importance in nutrition and economy, which makes them an alternative for the solution of hunger and malnutrition problems that are common in continents such as Africa (56–60).

### Economic and Gastronomic Importance

The gastronomy of Michoacán comprises the foodstuffs, culinary techniques, and the traditional dishes of the state. The diverse ecosystems have enabled the development of a culinary tradition that is varied, abundant, and millenary. It is the heritage of the pre-Hispanic people that lived there, such as the Purépechas, Nahuas, Mazahuas, and Mixtecos. As a result, Michoacán has the reputation of being one of the states with the greatest gastronomic culture in which insects along with corn have been one of the basic foodstuffs of Mexican cuisine since pre-Hispanic times. At present, they are still included in the diet due to the high protein content they have in diverse stages of development. This state culturally considers entomophagy as beneficial to both nutrition and the economy, with a significant commercial future.

In the state of Michoacán, as a strategy to make known the virtues of edible insects in all social strata, the students of the Biology Faculty of the Universidad Michoacana de San Nicolás de Hidalgo and of the Gastronomy School of the Universidad Vasco de Quiroga carried out a gastronomical exhibition in the city of Morelia, the state's capital, and prepared a great variety of insect-based dishes. This activity was supported both by the government and the productive sector, including the honorable city council of Morelia; the municipal departments of tourism, culture, and rural development; and the state's coordination committee for communication, ecotourism, e-fest extreme productions, and hyperlink publicity, as it is considered a promising commercial activity (46).

This exhibition hosted almost 2,500 visitors, a large-scale event that was widely publicized in Mexico and abroad by diverse television and radio networks and by some newspapers of the United States, Canada, and Latin America. It was visited by persons of different social classes, both male and female, and of different age groups that were able to taste and savor the dishes that were the attraction of the exhibition.

Detailed below are some forms of preparation used in Michoacán of some of the edible insects recorded. Order Orthoptera encompasses many phytophagous species that are pests, of which some are also predators; in this order, the nymphs and adults of grasshoppers are the Mexican edible insects most sought after, asked for, and commercialized in several states like Tlaxcala and Puebla, and they even have given rise to alimentary industries in Oaxaca. The grasshoppers (*Sphenarium*), before being prepared in diverse dishes, are dehydrated in a microwave oven to eliminate excess water and, if they are used in the preparation of a sweet dish, they are placed in a container with sugar and cinnamon, boiled, and then microwaved. With grasshoppers, they make stuffed sweet crepes with a chocolate covering, cheese pie with kiwi and strawberries, rice and milk, pizza, and *tacos* with tomato sauce. These grasshoppers are also consumed as an appetizer; they are toasted and seasoned with *piquing chili* or they are cooked so as to be eaten in *tacos* accompanied by *guacamole* or sauce. They are also prepared in a sauce made of *morita chili*, garlic, and *tomatillo* and are sold in the streets, fried and seasoned with lemon and chili. They are used in the design of delicious gourmet dishes.

For order Hemiptera, we see the following: The *Jumiles* (from the náhuatl, *xotlimilli*) are bug species pertaining to the family Pentatomidae that are considered to be a delicacy, where both nymphs and adults are consumed. The best known in the state is *Brochyymena tenebrosa*; these organisms can be eaten fried or ground to prepare a sauce and they may also be prepared in *enchiladas* (61). The *axayacatl* (a mix of eggs, nymphs and adults) is considered a delicacy since pre-Hispanic times and it is consumed toasted. The *cocopaches* (nymphs and adults) are eaten prepared in a sauce of chopped greens (tomatoes, onions, chili, and coriander) known as “pico de gallo” (rooster's beak).

For order Coleoptera, we see the following: This order includes the highest number of species in the Class Insecta, the adults of which receive diverse names, such as *mayates*, beetles, ladybirds, weevils, and *picudos*. They feed on both live and dead organic matter and, thus, they are important agricultural and forest pests; nevertheless, some of them are used in pest control. In Mexico, members of 22 families, 66 genera, and 119 species are consumed (11).

In the case of grub worms (*Phyllophaga*), they are cooked and filled with cheddar or mozzarella cheese, wrapped in a piece of bacon and accompanied by a cherry tomato, presented as sweet brochettes with kiwi, strawberry, and pineapple; as chocolate covered larvae; or as salted brochettes with broccoli, carrots, onions, and green pepper with a yellow dip with garlic; and as Chinese rolls with cream cheese and cucumber in a soy sauce. Depending on which flavor is selected when they are prepared, if it is salty they are fried in olive oil with salt and garlic, and if it is sweet with butter and sugar; and it is important to emphasize that the smell perceived during this preparation process is delightful, and it invites its savoring.

For the order Lepidoptera, we see the following: It encompasses the insects known as butterflies and moths, and among them, the edible ones that are most popular are the white and red maguey worms that are distributed in all states of Mexico where this plant grows. The *cupiches* (larvae), *huenches, conduchas*, or *chamas* (pupae) are toasted in a *comal* and are eaten in *tacos* accompanied by hot sauce. Red and white maguey worms (larvae) are eaten in *tacos*.

For order Hymenoptera, we see the following: This order is equally one of the most numerous species, which are known by the common names of wasps, bees, and ants, that are beneficial, and some have been domesticated, as is the case of the bee *Apis mellifera*, while others are involved in pollination and biological control.

Bee larvae (*Apis*) are prepared with chantilly cream cheese filled strawberries, the strawberries are cored and filled with the mix of the cheese, cream, and larvae; they can also be added to rum-flamed bananas or to the custard used for filling pies; larvae are added directly to the dishes, giving them a sweet and pine nut like flavor. The reproductive adults of the *chicatana* ants are consumed fried and roasted and in sauces; another preparation form is to toast them and then grind them in a *molcajete* with chili, garlic, and salt so as to be eaten in *tacos*.

The visitors and chefs who tasted these dishes found the diverse gastronomic presentations made with these insects palatable. They supported the promotion of this type of research and outlined that they would potentially include insects in their diet. They outlined that the aesthetic presentation of the dishes was encouraging, and many proposed that they would be the food of the future, an idea also put forward by Ramos-Elorduy (62).

In relation to insect gastronomy, there exists in Morelia, Michoacán, a catering micro-company called “Bichus Delicious” (natural protein source), whose founder, the entrepreneur Janette Lagunas Rayas, has published that the market of insect consumption is growing slowly, but it is nevertheless growing (63). This company is focused mainly on the distribution of edible insects that are consumed with mescal made in Michoacán. “They are appetizer grasshoppers that are seasoned with salt, lemon, chili and a bit of garlic, also salt that has been seasoned with maguey worms,” says the founder, who points out that they also sell salts flavored with grasshoppers and maguey worms (larvae), grasshopper chips, peanuts with grasshoppers, appetizers that accompany drinks like mescal and tequila, a mixed appetizer of peanuts with grasshoppers and *chinicuil* (larvae), grasshopper flour, grasshopper and *chinicuil* chocolates, and caramelized grasshoppers. She says that the acceptance of these foodstuffs has been growing in Morelia, the capital city of the state, especially in the health sector due to the nutritional virtues of insects that, as we have already mentioned, are widely documented in the scientific literature, both in Mexico and worldwide. This firm also organizes training courses in rural zones where they show how to collect, clean, and prepare them for their sale, so as to ensure that they are innocuous in the processed products. This activity has become the source of income of many persons who are now devoted to collecting grasshoppers for human consumption. This is proof that this activity enables them to obtain a significant income (64, 65), for example, a bag of dehydrated grasshoppers containing 20 g costs 35 pesos that are ~US$1.74; therefore, for the sale of 1 kg, 87 dollars are obtained. They are currently working with some mescal producers, using as a complement the grasshoppers and the salts, and that these commercial activities are profitable. Grasshoppers have an important potential market through products such as “*salpul*í*n*,” a mix of salt and ground grasshoppers, a “grasshopper seasoning,” which is a combination of salt, spices, and ground grasshoppers that can be added to meats, soups, and other preparations such as the “jumping cookies” made with flour (66). Some farmers of Tarímbaro, Michoacán, have even industrialized grasshoppers as a method of pest control of their crops and as an alternative commercial protein-based food (67). In this case, instead of buying insecticides for pest control, they have opted for training 60 farm hands in the new control methods with alimentary objectives, thus reducing the pollution that arises from applying insecticides.

Other insects that have economic importance in diverse ways include the bees known as Uauapu bees (eggs, larvae, and pupae). Honey is sold in local and regional markets. Honey is considered a very rich foodstuff both for its flavor as well as for its nutritional properties; it is consumed because people like it and it is considered a privileged foodstuff. The honeycomb is also sold commercially and eaten in sliced pieces, and the larvae are consumed as well (45). Honey is used in therapeutic procedures, for example, it is used as an antiseptic and healing agent. Pollination: The importance of the uauapu bee in the pollination and reproduction of both wild and cultivated plants has been widely recognized, for example, in the following localities: Cherán, Urapicho, Angahuan, Zipiajo, Comachué, Uricho, Cheran, Cocucho, Charapan, and Tanaco (45). In brief, the “uauapu” complex is important in the life of the Purépecha communities, especially with respect to their alimentary, medicinal, and agricultural culture (68). Despite the benefits and the potential commercial prospects of this honey, there are a number of challenges, as it is harder to find the bees and honeycombs near villages due to the following: forest fires, forest deterioration due to felling, competition with the European introduced bees, changes in soil vocation, expansion of the farming frontier and the urban footprint, and use of nicotinic insecticides and pesticides; all these factors are related with the bee collapse syndrome that accounts for the disappearance of 40% of the beehives in the state (64, 69).

## Conclusions

In the state of Michoacán, Mexico, 69 species of edible insects in their different stages of development are consumed. The localities in which the greatest number of species are consumed are: Charapan (10 species) Jungapeo (7), Pátzcuaro (6), San Pedro Tarímbaro (6), Neocupétaro (6), and Cherán (6).

Anthropo-entomaphagy persists in Michoacán due to its rural population that, despite the influence of miscegenation and the introduction of other alimentary habits, has kept its traditional knowledge about the ecology, distribution, management, and consumption of edible insects as these arthropods have been part of culture since pre-Hispanic times. They have been a significant aspect of Mexican cuisine and diet for centuries. Today, many chefs have incorporated entomophagy into Mexican haute cuisine, developing dishes that, although exotic and expensive to certain social strata, are nevertheless increasingly more acceptable and consumed in different restaurants and markets.

However, it must be emphasized that even with the number of species registered in the state, anthropo–entomophagy is not very popular in the big cities and municipal heads; they used to be consumed in rural zones in which they are collected, prepared, and eaten; likewise, we observed that this activity is very much appreciated by the elders and ignored by the young. This is due to the large quantity of modern foodstuffs which the latter tend to consume as part of their diets.

Finally, we can say that this pre-Columbian legacy prevails, and we ascertain that edible insects are an essential part of our alimentary culture and an element of identity that generates nutritional, medicinal, and economic benefits to those that practice entomophagy in the Mexican Republic.

## Data Availability Statement

The raw data supporting the conclusions of this article will be made available by the authors, without undue reservation.

## Author Contributions

Both authors participated in the field research, writing, and final review of the article.

## Conflict of Interest

The authors declare that the research was conducted in the absence of any commercial or financial relationships that could be construed as a potential conflict of interest.
